# Hydrodynamic Kelvin–Helmholtz instability on metallic surface

**DOI:** 10.1038/s41598-023-29810-7

**Published:** 2023-02-15

**Authors:** Xi Wang, Xiao-Mian Hu, Sheng-Tao Wang, Hao Pan, Jian-Wei Yin

**Affiliations:** Institute of Applied Physics and Computational Mathematics, 100094 Beijing, People’s Republic of China

**Keywords:** Fluid dynamics, Surfaces, interfaces and thin films

## Abstract

Kelvin–Helmholtz instability on metallic surface is relevant to intense oblique impact in many physical processes such as explosive welding, Inertial Confinement Fusion and planetary impact events. Evolution of instability results in the formation of wavy morphology leading to material bonding or even mixing. However, mostly due to lack method to describe the dynamic behavior, instability mechanism controlled by elastoplastic properties of metal remains elusive. Here, we introduce a theory to reveal the evolution characteristics aroused by tangential velocity. Our simulations find that the unstable metallic surfaces exhibit amplitude growth and tangential motion by overcoming the depression of yield strength to generate wavy morphology. For diverse loading velocities, corrugated surfaces and material properties, an instability boundary distinguishes all unstable evolutions. Our analytical method with scale-independent variables reproducing numerical findings reveals plentiful characteristics of instability in strength materials. For designed loading velocities and material in oblique impact experiment in laboratory, the property of corrugated surfaces becomes an important factor to determine instability evolution.

## Introduction

Kelvin–Helmholtz instability(KHI)^1,2^ due to shear at metallic surface remains scarcely understood, which especially deserve to interpret as metal suffering intense oblique impact in High Velocity Impact Welding (HVIW)^3–5^, Inertial Confinement Fusion (ICF)^6,7^, planetary impact events^8–10^, etc. The wavy structures aroused by tangential velocity jump at the instance of surface collision with angles indicate material bonding or even potential mixing^5,8^. Although KHI between fluids are studied extensively^11,12^, characteristics of KHI evolution associated with depression effects of elastic–plastic (EP) properties of metal^13^ merit thoroughly digging.

Detection of KHI on metallic surface is a serious challenge due to practical difficulties of sustainment of high-speed shear flow in experimental facilities^14^. The characteristics of wavy morphologies are usually discussed with the help of high velocity oblique impact experiment whose results can only be imaged at the end of experiments which do not reveal the evolution processes^3,4,15,16^, not mentioning another issue of recovering sample without severe fragmentations under high velocity loading^17^. Although oblique impact processes can be exhibited by computer simulations, besides acquiring adequately fine mesh distributions, the accuracy of calculations is largely determined by different arithmetic of capturing material interface^15,18–20^. For KHI on metals, it is surprising that relevant simulations have not been shown for now but only theoretical analysis with traditional normal mode method which merely presents growth rate and involves impossibility of analytical treatments due to nonlinear governing equations and nonlinear constitutive relations of metal^5,18^. As a result, we especially lack the descriptions of evolution characteristics of metallic perturbed surface under the operation of tangential velocity discontinuity.

For the purpose of investigating surface behavior of KHI on solid, we have proposed a theoretical analysis with a potential flow method to describe the growth rate and amplitude evolution by analytical formulas^21^. The properties of resisting shear deformation of solid material influence the instability evolution of surface as flushed by tangential flow. The amplitude growth is prevented by EP properties of solid to become behavior of oscillating around. Although the depression effect of EP properties has been detected in the amplitude evolution, it is interesting that the growth rate is the same as KHI for different ideal fluids, i.e. $k\sqrt{{\rho }_{1}{\rho }_{2}{u}_{0}^{2}}/({\rho }_{1}{+\rho }_{2})$, which is always positive to indicate continuous growth of amplitude. The traditional method to estimate whether the surface is stable or unstable by growth rate^18,22^ seems invalid for solid. Besides, the relation between EP transition and instability evolution can not also be exhibited by growth rate and amplitude. In present work, we attempt to illuminate a method to estimate whether instability develop named instability boundary and to explain the effect of EP transition on instability by EP division.

**Figure 1:**
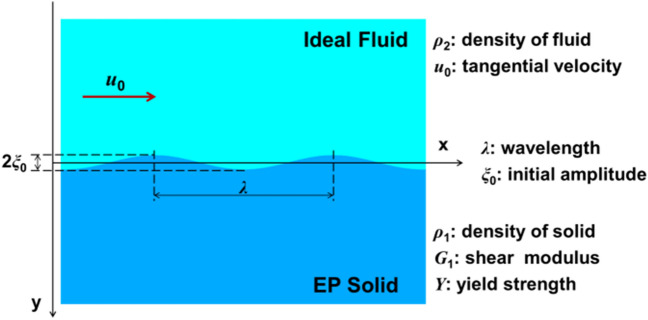
The configuration of ideal fluid flowing over a perturbed surface of perfectly EP metal. The solid with density *ρ*_1_ is quiescent in the 2D Cartesian coordinate system and the fluid with density *ρ*_2_ has a constant tangential velocity *u*_0_ in *x* direction. The initial perturbation has a cosinusoidal form *ξ*_0_cos*kx* with periodic wavelength *λ* and amplitude *ξ*_0_. Both thicknesses *h*_1_ and *h*_2_ of materials are large enough to make sure approaching infinite medium, i.e. *kh*_1_ >> 1 and *kh*_2_ >> 1.

Here, we consider the instability for the configuration of an ideal fluid with constant tangential velocity *u*_0_ gliding over a quiescent perfectly EP solid (Fig. 1). For simplicity our discussion is restricted in two-dimensional plane with *y*-axis perpendicular to flow direction *x*. The small perturbation can be represented by *η*(*x*,*t*) = *ξ*(*t*)*e*^*ikx*^ where *ξ*(*t*) is amplitude with initial value of *ξ*(0) = *ξ*_0_ and *k* = 2*π*/*λ* is wave number for wavelength *λ*. The metal and fluid may commonly have different densities of *ρ*_1_ and *ρ*_2_. In this system, *u*_0_ is the inducement of instability, yet the surface may be stable under the suppression of constant shear modulus *G*_1_ and constant yield stress *Y* before and after plastic deformation. Then several dimensionless variables which characterize a KHI system are defined: *A*_*T*_ = (*ρ*_1_ - *ρ*_2_) / (*ρ*_1_ + *ρ*_2_) is Atwood number; *M*_0_^2^ = *ρ*_1_*u*_0_^2^/*G*_1_ is Mach number; *z* = *ξ*(*t*)/*ξ*_0_ is growth factor; *τ* = *tku*_0_, $\widehat{\lambda }\hspace{0.17em}$= 2*πξ*_0_/*λ* and $\widehat{Y}\hspace{0.17em}$= *ρ*_1_*u*_0_^2^/*Y* are respectively dimensionless time, wavelength and yield strength.

We first execute extensive numerical calculations to reveal the time evolutions of metallic perturbations. The simulations perform the generation of various wavy morphologies displaying continuous growth of amplitude and tangential motion of wave crest which implies system instability. Based on the characteristics of instability evolution, an instability boundary separating all stable and unstable cases is achieved, and EP division splitting all cases without and with plastic behaviors is also obtained. Furthermore, a theoretical method is carried out to disclose analytical expressions of instability boundary and EP division which are evaluated by simulation findings. The identical results show that the seemingly complex instability can be quantitatively described by the defined scale-independent variables to elucidate the characteristics of instability evolution by the suppression of shear modulus and yield strength from a straightforward perspective. That makes the theory a potential versatile method to estimate similar KHI system on any other strength medium whether the surface is unstable to form wavy patterns after high intense oblique impact.

## Results

### Numerical simulations

The KHI configuration in Fig. 1 is simulated by finite element method^23^ (see “Methods” section). At initial moment, water with constant tangential velocity closely contacts to perturbed copper surface. Scales of variables are chosen by consulting oblique impact experiments^24,25^.

**Figure 2:**
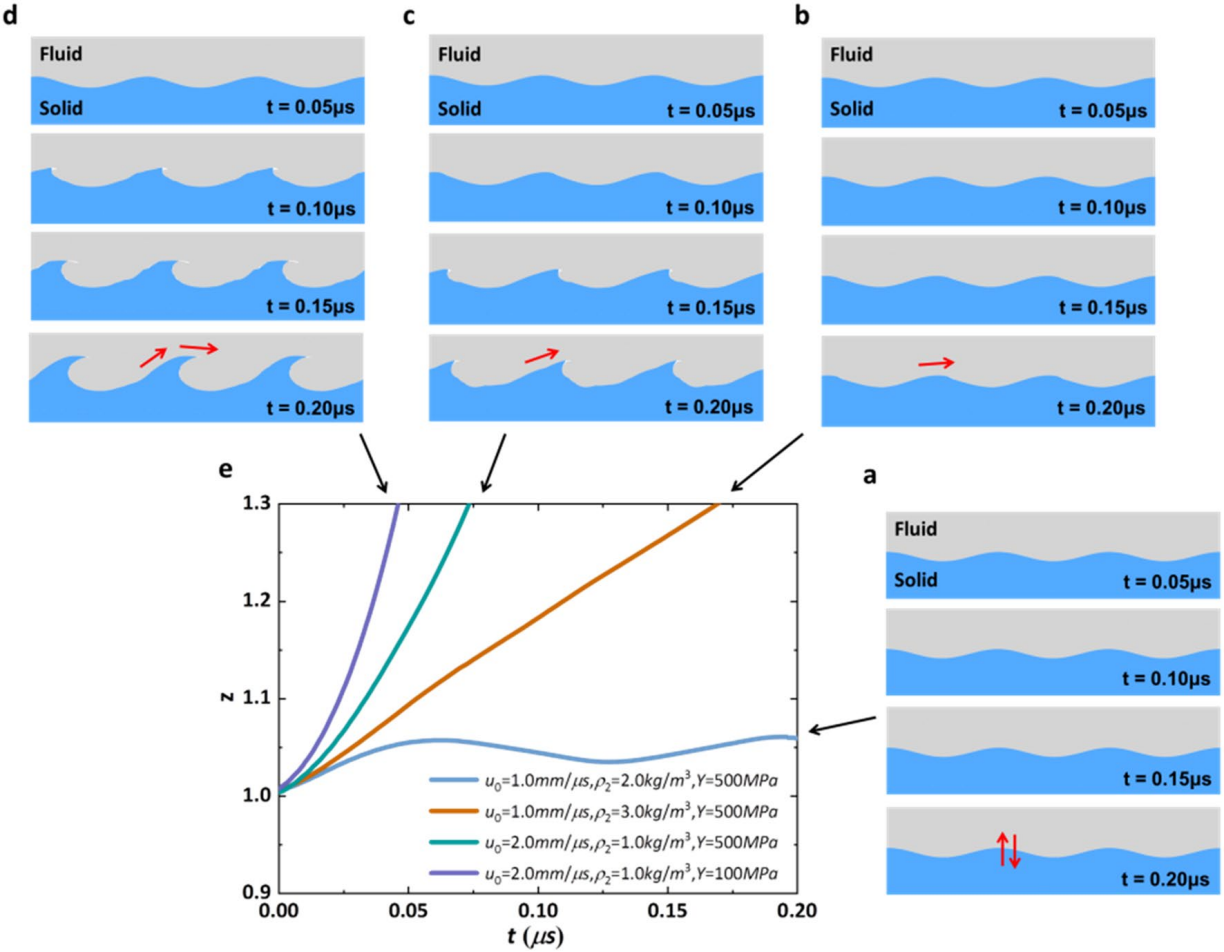
Four cases of surface temporal evolutions. (** a**–**d**) Maps of surface temporal morphologies of quiescent Cu plate surface (blue) flowed by ideal fluid H_2_O (grey) with tangential velocity *u*_0_, as calculated by finite element methods^23^. Each Cu plate has fixed density *ρ*_1_ = 8.9 kg/m^3^, fixed shear modulus 39.39 GPa and the same initial perturbation with 250 μm wavelength and 10 μm amplitude. The four cases are obtained by varying tangential velocity, fluid densities and yield strength of Cu for case a* u*_0_ = 1.0 mm/μs, *ρ*_2_ = 2.0 kg/m^3^, *Y* = 500 MPa, case (**b**) *u*_0_ = 1.0 mm/μs, *ρ*_2_ = 3.0 kg/m^3^, *Y* = 500 MPa, case (**c**) *u*_0_ = 2.0 mm/μs, *ρ*_2_ = 1.0 kg/m^3^, *Y* = 500 MPa and case (**d**) *u*_0_ = 2.0 mm/μs, *ρ*_2_ = 1.0 kg/m^3^, *Y* = 100 MPa. Surface morphologies of four cases at time 0.05 μs, 0.1 μs, 0.15 μs, 0.2 μs are given respectively. The red arrow in each surface at time 0.2 μs is the schematic of surface motion direction. (**e**) The growth factor *z* of each case is extracted from simulation.

After abundant calculations, we performed four typical time evolutions of metallic surfaces including stable and unstable cases. Due to the depression of shear modulus *G*_1_ and strength *Y*, the stable surface maintains similarity to the initial perturbation as time passing (Fig. 2a), and the amplitude presents oscillating behavior around a small range in vertical direction (Fig. 2e). Under the operation of velocity in *x* direction, the unstable evolutions perform *y* direction growth accompanying tangential direction motion such as slight tangential shifting (Fig. 2b), visible tangential movement (Fig. 2c), or surface rolling up (Fig. 2d). The growth factors of unstable wavy patterns all exhibit the trends of increasing with different rates (Fig. 2e). By the way, the wavy morphologies of unstable surfaces in our simulations at time 0.15 μs and 0.2 μs (Figs. 2b–d) are similar to those observed in oblique impact experiments, including different tangential movement behavior and curling structure^24–28^.

**Figure 3:**
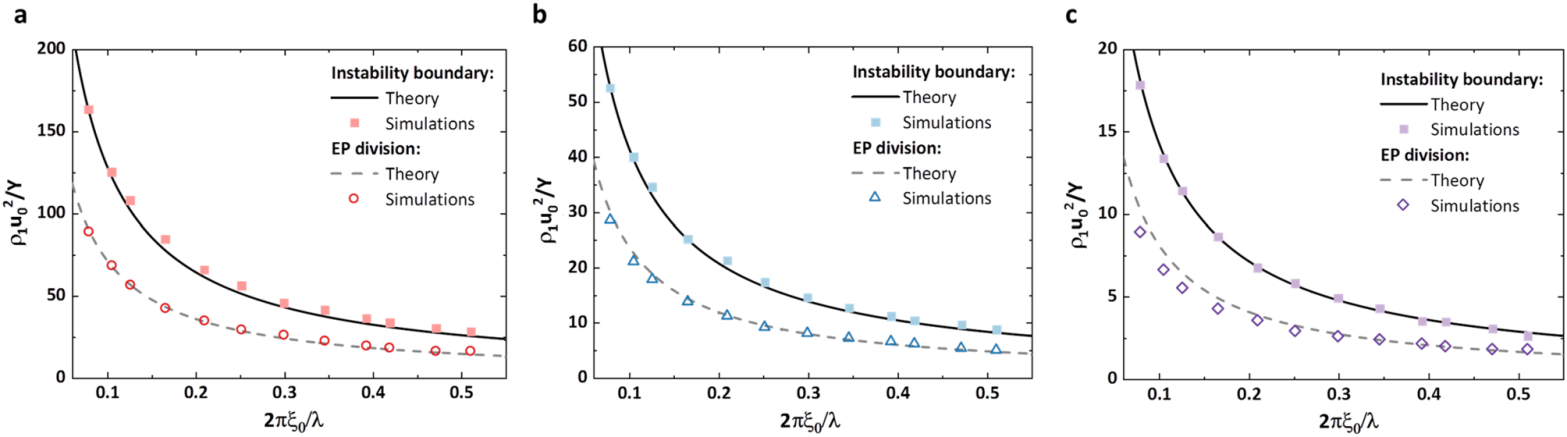
Instability boundary and EP division by simulations and theory. Three combinations of *A*_*T*_ and *M*_0_ are calculated i.e. (**a**) *A*_*T*_ = 0.7980 and *M*_0_ = 0.4754, (**b**) *A*_*T*_ = 0.4958 and *M*_0_ = 0.3803, (**c**) *A*_*T*_ = 0.0 and *M*_0_ = 0.2377. The axis 2*πξ*_0_/*λ* and *ρ*_1_*u*_0_^2^/*Y* in the figures are $\hat{\lambda }$ and $\hat{Y}$. Solid point and hollow point in each figure represents simulation results of instability boundary and EP division respectively. Solid line and dash line means results by theory for instability boundary and EP division. The results with identical *A*_*T*_ and *M*_0_ by numerical simulation and analytical theory are plotted in one figure for comparison.

Due to obvious disparity of amplitude development, we find a boundary to partition stable and unstable evolutions for all variable combinations (Fig. 3). Different dimensionless variables *A*_*T*_, *M*_0_, $\widehat{\lambda }$ and $\widehat{Y}$ in simulations are obtained by changing fluid density, tangential velocity, initial amplitude, wavelength, shear modulus and yield strength respectively (see Supplementary). According to stable oscillation and continuous increase of growth factors, the boundary is achieved by fixing $\widehat{\lambda }$ and varying $\widehat{Y}$ with enough point densities to approach the position of instability margin. Then, adopting the same procedure to more $\widehat{\lambda }$ values, a line can be drawn to split the domains. As shown in Fig. 3 with different *A*_*T*_ and *M*_0_, the areas containing all variable combinations below and above instability boundary respectively indicate stable and unstable surface. Similarly, EP division partitioning amplitude motion with and without plastic behavior is also achieved. The area below EP division implies elastic deformation, as plastic behavior above the division line. We also observe that EP division is beneath instability boundary hinting that continuous growth of perturbation must experience plastic transformation to overcome the resistance of deforming from the initial perturbation.

### Theoretical analysis

Instability analysis starts from the governing equations of continuity and momentum with the assumptions of incompressible and irrotational flow. Potential flow method is adopted to represent the velocity field which should be continuous in the normal direction of material interface with the perturbation of *η*(*x*,*t*) = *ξ*(*t*)*e*^*ikx*^. Then, the motion equation for describing evolution of interface amplitude *ξ*(*t*) is achieved with the condition of force equilibrium in the normal direction of material interface. We give a specific case of the process of establishing the instability analysis in “Methods” section. With perfectly EP properties of solid and Cauchy stress of viscous fluid, the motion equation of interface between EP solid and viscous fluid is obtained. The instability of solid is focused in the present study to ignore the viscosity of fluid. The motion equation of amplitude is changed to be a dimensionless form (Eq. (21) in “Methods” section)

1 $$\ddot{z}+2{M}_{1}i\dot{z}=\left\{\begin{array}{c}\Lambda -\left(\Lambda -{M}_{2}\right)z,\hspace{1em}\hspace{1em}z\le {z}_{p}\\ {M}_{2}z-{\rm X},\hspace{1em}\hspace{1em}\hspace{1em}\hspace{1em}\hspace{0.33em}z>{z}_{p}\end{array}\right.$$

where *z*_*p*_ is growth factor at when EP transition takes place and

2 $${z}_{p}=\frac{\rm X}{\Lambda }+1,\hspace{1em}{M}_{1}=\frac{1-{A}_{T}}{2}\hspace{0.33em},\hspace{1em}{M}_{2}=\frac{1-{A}_{T}}{2}\hspace{0.33em},\hspace{1em}\Lambda =\frac{1+{A}_{T}}{{M}_{0}^{2}}\hspace{0.33em},\hspace{1em}{\rm X}=\frac{e}{2\sqrt{3}}\frac{1+{A}_{T}}{\widehat{\lambda }\widehat{Y}}\hspace{0.33em}.$$

The symbol Λ and Χ contain influences of shear modulus and yield strength represented by dimensionless variables *M*_0_ and $\hat{Y}$. In Eq. (1) the first branch controls amplitude motion with elastic behavior before growth factor *z* arrives at *z*_*p*_ beyond which plastic deformation occurs to depict the amplitude behavior by the second branch. Starting from Eq. (1) with mathematical deriving (see “Methods” section), we disclose the analytical formulas of instability boundary (Eq. (32) in “Methods” section)

3 $${\widehat{Y}}_{IB}=\frac{e}{2\sqrt{3}}\frac{1+{A}_{T}}{\Lambda \widehat{\lambda }}\frac{\Lambda -{M}_{2}}{{M}_{2}-{x}_{p}{e}^{-i{M}_{1}{\tau }_{p}}}\hspace{0.33em},$$

and EP division (Eq. (38) in “Methods” section)

4 $${\widehat{Y}}_{EP}=\frac{e}{2\sqrt{3}}\frac{1+{A}_{T}}{\Lambda \widehat{\lambda }}\frac{\Lambda -{M}_{2}}{{M}_{2}\left(1+{e}^{-i{M}_{1}{\tau }_{e}}\right)}$$

The specific formulations of *x*_*p*_ ,*τ*_*p*_ and *τ*_*e*_ (see Eqs. (30), (28) and (36) in “Methods” section) are relative to *M*_1_, *M*_2_ and Λ, therefore instability boundary and EP division with the relation of $\widehat{Y}$= *f* ($\widehat{\lambda }$) are determined by *A*_*T*_ and *M*_0_. Lines calculated by Eqs. (3) and (4) with same dimensionless variables as simulations are also plotted in Fig. 3 which show identical results.

Furthermore, we perform more properties about instability boundary, EP division and amplitude evolution (solutions of Eq. (1) in “Methods” section) by our theory to understand the instability behavior.

**Figure 4:**
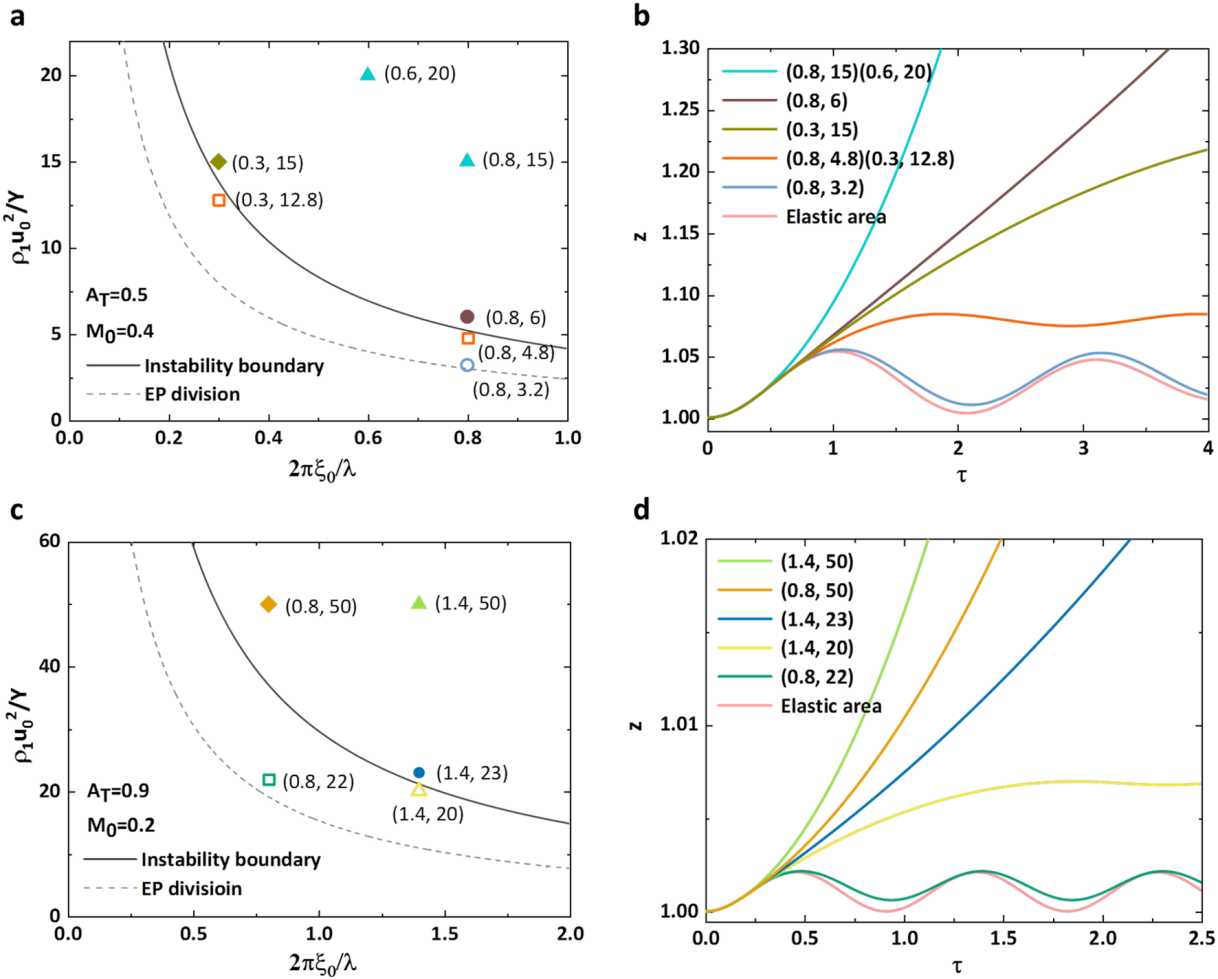
Two groups of instability boundary, EP division and growth factor by theory. (**a**,**b**) The first group of instability boundary, EP division and growth factor for arbitrarily picked points with *A*_*T*_ = 0.5, *M*_0_ = 0.4. (**c**,**d**) The second group with *A*_*T*_ = 0.9, *M*_0_ = 0.2. The solid points meaning picked variable combinations locate above instability boundary, and the hollow points are between instability boundary and EP division. $\hat{Y}$ and $\hat{\lambda }$ values of each point are marked in bracket after point. Some of them have the same $\hat{\lambda }$, some have the same $\hat{Y}$. For the points with the same shape and color, their $\hat{Y}\hat{\lambda }$ is identical. The color of the curve of growth factor z in (**b**) and (**d**) figures are completely the same as point color. The line labeled as "Elastic area" is the growth factor for the region beneath EP division, and other lines are labeled with corresponding $\hat{Y}$ and $\hat{\lambda }$ values in bracket.

Instability boundary and EP division divide $\widehat{Y}$ vs $\hat{\lambda }$ field into three parts. Some points in each part are picked to perform amplitude time evolutions (Fig. 4). Figure 4a plots instability boundary and EP division for *A*_*T*_ = 0.5 and *M*_0_ = 0.4. The area below EP division means amplitude motion only with elastic behavior whose growth factor is also determined by *A*_*T*_ and *M*_0_ (Eq. (39a) in “Methods” section), therefore all parameter combinations in the area below EP division possesses the same time evolution which is controlled by *G*_1_ leading to vibrate in a small range (Fig. 4b).The area between instability boundary and EP division implies the amplitude is stable with plastic deformation. The growth factor initially elastically oscillate to exceed *z*_*p*_ to plastic stage, then is suppressed by *Y* to a maximum value and oscillates around (Eq. (39b) in “Methods” section). For the regions above instability boundary, the growth factor also elastically vibrates to plastic stage, but the effect of *Y* is evidently weak as shown continuous increase of amplitude in Fig. 4b (Eq. (39c) in “Methods” section). Except for the features illustrated above, some others are also detected, such as growth factor exhibit more stable motion as $\widehat{Y}$ or $\widehat{\lambda }$ lessen, and identical $\widehat{Y}\widehat{\lambda }$ specifies the same evolution in plastic stage (Eqs. (39b) and (39c) in “Methods” section). The corresponding values of $\widehat{\lambda }$ and $\widehat{Y}$, EP state and surface state are summarized in Tables 1 and 2 for Fig. 4. Besides, we also give instability boundary, EP division and amplitude evolution for another *A*_*T*_ = 0.9 and *M*_0_ = 0.2 (Fig. 4c,d). The regularity of surface development are similar to those with *A*_*T*_ = 0.5 and *M*_0_ = 0.4 except that the range of oscillation is obliviously narrower.

**Table 1:** Details for results in Fig. 4a,b.

$\widehat{\lambda }$	$\widehat{Y}$	$\widehat{\lambda }\widehat{Y}$	State of deformation	State of stability
Region below EP division	Region below EP division	Region below EP division	Elastic behavior	Stable
0.8	3.2	2.56	Plastic behavior	Stable
0.8	4.8	3.84	Plastic behavior	Stable
0.3	12.8	3.84	Plastic behavior	Stable
0.3	15	4.5	Plastic behavior	Unstable
0.8	6	4.8	Plastic behavior	Unstable
0.8	15	12	Plastic behavior	Unstable
0.6	20	12	Plastic behavior	Unstable

**Table 2:** Details for results in Fig. 4c,d.

$\widehat{\lambda }$	$\widehat{Y}$	State of deformation	State of stability
Region below EP division	Region below EP division	Elastic behavior	Stable
0.8	22	Plastic behavior	Stable
1.4	20	Plastic behavior	Stable
1.4	23	Plastic behavior	Unstable
0.8	50	Plastic behavior	Unstable
1.4	50	Plastic behavior	Unstable

**Figure 5:**
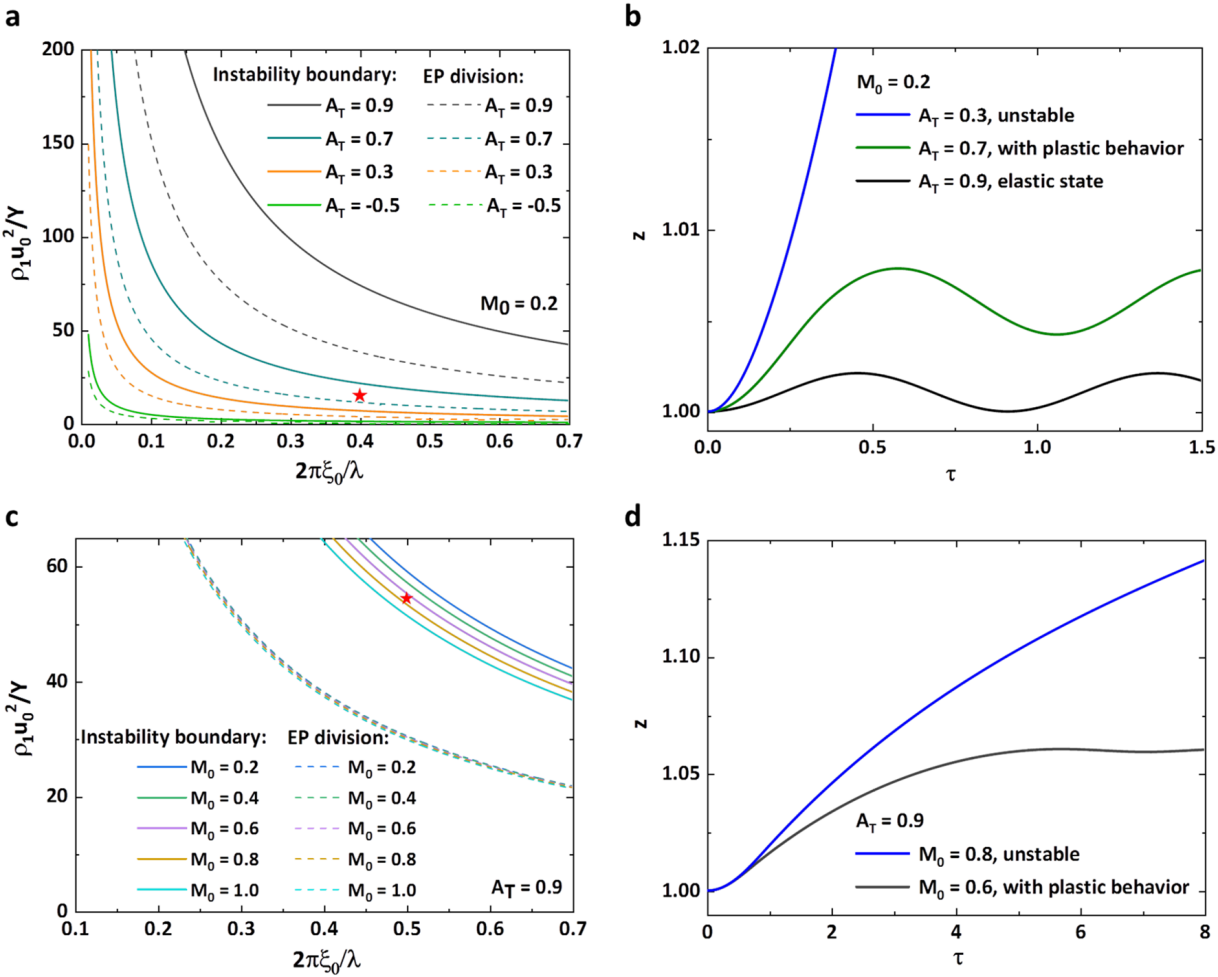
Influences of *A*_*T*_ and *M*_0_ on instability boundary and EP division. (**a**) For a fixed *M*_0_ = 0.2, four groups of instability boundary (solid line) and EP division (dash line) with *A*_*T*_ = 0.9, 0.7, 0.3, − 0.5 are plotted. A point between instability boundary and EP division of *A*_*T*_ = 0.7 is designated with red star. (**b**) The growth factor of red star in figure (**a**) as *A*_*T*_ varying. (**c**) For a fixed *A*_*T*_ = 0.9, five groups of instability boundary (solid line) and EP division (dash line) with* M*_0_ = 0.2, 0.4, 0.6, 0.8, 1.0 are shown. Also a red star between *M*_0_ = 0.6 and 0.8 is designated. (**d**) The growth factor of red star in figure (**c**) as *M*_0_ varying.

Due to *A*_*T*_ and *M*_0_ deciding instability boundary and EP division, we also illustrate the influences. By decreasing *A*_*T*_ gradually for *M*_0_ = 0.2 (Fig. 5a), instability boundary and EP division are simultaneously moving close to the coordinate axis which show the area of amplitude growth and plastic motion are enlarged. For a fixed combination of $\hat{Y}$ and $\hat{\lambda }$ which is designated in Fig. 5a, as varying *A*_*T*_ the point locates at different areas. For *A*_*T*_ = 0.9, the point is in the elastic area and the corresponding amplitude elastically oscillates (black line in Fig. 5b). As *A*_*T*_ = 0.7, the point is between instability boundary and EP division and the growth is controlled by yield stress (green line in Fig. 5b). After *A*_*T*_ = 0.3, the point is in the instability area to exhibit a continuous increasing amplitude (blue line in Fig. 5b). Besides, we also find that as *A*_*T*_ decrease the area between instability boundary and EP division diminishes which indicates that the surface is unstable once plastic transformation occurs for small *A*_*T*_. Then, the features of instability boundary and EP division affected by *M*_0_ are plotted in Fig. 5c,d. The influences of *M*_0_ are not that evident especially on EP division and as increasing *M*_0_ instability boundary presents appreciable movement in the direction of approaching to axis. For a point between instability boundary of *M*_0_ = 0.6 and 0.8, the growth factor must undergo plastic deformation, and the amplitude is stable for *M*_0_ = 0.6 (black line in Fig. 5d) and unstable for *M*_0_ = 0.8 (blue line in Fig. 5d). It seems that the state of amplitude evolution of variable combinations near the instability boundary is sensitive to the change of *M*_0_.

The prominent characteristic of solid is the ability to resist shear deformation. Solid body deforms when it is subjected to external forces. If the deformation is reversible, i.e. the deformation vanishes instantaneously as soon as external forces are removed, this type of deformation is elastic. If the deformation is permanent, i.e. solid yields, solid deforms with plastic behavior. The resistance to shear deformation obviously determines the instability of solid surface which are shown in the above results.

Those results show that a whole picture of KHI in solid are becoming clear through instability boundary, EP division and evolution of growth factor which are denoted by dimensionless variables *A*_*T*_, *M*_0_, $\widehat{\lambda }$ and $\widehat{Y}$. The instability boundary and EP division are built in the plane of $\widehat{\lambda }$ and $\widehat{Y}$ which are divided into three parts. The relative position of instability boundary and EP division is that instability boundary locates above EP division in $\widehat{\lambda }$ and $\widehat{Y}$ plane which indicates physical insights of the characteristics of instability evolution. The first part in $\widehat{\lambda }$ and $\widehat{Y}$ plane is the region below EP division in which solid does not reach the yield point and the surface growth is restrained by shear modulus. The amplitude shows vibration around a small range due to resisting shear deformation in elastic stage. The morphology of surface may be reversible to original state if the inducement of instability disappears. The second part in $\widehat{\lambda }$ and $\widehat{Y}$ plane is the region above EP division and beneath instability boundary in which yield occurs and the surface growth is controlled by strength. In the second part, elasticity of solid can not suppress amplitude growth and deformation becomes large to plastic stage. Yet, the continuous growth of amplitude is restrained by yield strength after EP transition. Therefore, the characteristics of amplitude evolution contain three processes including elastic vibration to plastic deformation, growth suppressed by strength and vibration around. The morphology of surface may be permanent if the inducement of instability disappears. Those two parts constitute all cases of stable surfaces. The third part in $\widehat{\lambda }$ and $\widehat{Y}$ plane is the region above instability boundary in which surfaces are unstable with plastic deformation. In the third part, the inducement of instability drives the surface deforming from elastic stage to plastic stage, and strength can not prevent increase of amplitude. The surface evolution exhibits large deformation to form wavy morphology. Although, instability boundary and EP division are affected by *A*_*T*_ and *M*_0_ with different degrees, the above physical regularity of instability evolution does not change. According to the analysis, it is found that EP transition is the necessary condition to amplitude growth to form wavy morphology, which imply that the minimal energy required to engender effective bonding or mixing between materials after oblique impact must exceed the energy leading to plastic deformation.

## Discussion

For KHI system we discussed above, the simulations demonstrate that the continuous growth of amplitude of metallic surface in early stage by overcoming the depression effects of EP properties is a signature of dynamic instability to form wavy morphology, and there is a boundary to distinguish all stable and unstable variable combinations for diverse velocities, perturbation geometries (initial amplitude and wavelength) and material properties (densities, shear modulus and yield strength).

Instability boundary and EP division predicted by our analytical formulas are intriguingly consistent with those obtained by simulations, and the model identifies dimensionless variables *A*_*T*_, *M*_0_, $\widehat{\lambda }$ and $\widehat{Y}$ as characteristic parameters to describe surface instability. The analytical results also quantitatively illustrate how the evolution processes are affected by those dimensionless variables from plentiful perspectives. Since our mathematical formulas do not include scale-independent terms, the model appears to be able to predict evolutions for broader KHI systems not only cases we discussed here.

Comparing the late-time morphology by our simulations with that after oblique impact experiment^24–28^, the characteristics of metallic wavy pattern are similar including amplitude growth and tangential motion which reproves that indeed the formation mechanism of wavy interface is the evolution of KHI^5,25^. The configuration in our study resembles the situation of a strength material collided with angle by a metal undergoing thermal softening^18^. Tangential velocity is decomposed from impact velocity according to impact angle between impactor and target. By extracting perturbation (e.g. surface roughness)^24,25^ and material properties of impactor and target, dimensionless variables of the configuration are easily calculated. Smaller *A*_*T*_ and larger *M*_0_ mean an impactor with larger density and velocity for the same target. Smaller $\widehat{\lambda }$ and $\widehat{Y}$ corresponds to a higher strength target with more gentle perturbation. We can predict the surface evolution state through locating the position between calculated ($\widehat{\lambda }$,$\widehat{Y}$) and instability boundary above which means wavy morphology formation. Thus, the presented theoretical method offers a potential versatile tool over a broad range of scales to help estimate the evolution of metallic surface after oblique impact to explain collisional bonding or even mixing^29,30^. For a more comprehensive evolution between two EP metals, the characteristics require further investigations.

The common assumption of material constitutive properties recognizing scaling strength linearly with the shear modulus^31,32^ and the constitutive behavior at high pressures^33,34^ indicate the connection between shear modulus and yield strength. For a designed condition of dynamic loading (tangential velocity) and samples (densities, shear modulus and yield strength), *A*_*T*_ , $\widehat{Y}$, *M*_0_ except $\widehat{\lambda }$ (initial amplitude and wavelength) can be roughly estimated, namely the instability state of surface are determined by the properties of perturbation including origin and dimension which are another complex problems referring to machining techniques, material microstructures etc. The wavy morphology with plastic deformation after oblique impact takes as evidence of bonding between dissimilar materials^29,30^, therefore perturbation becomes a crucial factor of effective bonding.

## Methods

### Simulation details

Numerical simulation taken in this paper is a 2D Lagrange finite element method which has been adopted to simulate dynamic impact and surface instability frequently^18,24^. The advantage of Lagrange method of capturing material interface is utilized to obtain a clear surface of solid. The surfaces of materials are initially contact and defined by a sliding_only contact which is a two-surface method. The details of framework and reliability of the Lagrange method is performed in previous study^21^.

The solid and fluid are both simulated with EOS of the Mie-Grüneisen state with a coefficient *γ* = *ρ*_0_*γ*_0_/*ρ* where *γ*_0_ is a parameter characteristic and *ρ*_0_ is the initial density. The relation between shock velocity *v*_*s*_ and particle velocity* v*_*p*_ is *v*_*s*_ = *c*_0_ + *sv*_*p*_ where* c*_0_ is the bulk sound velocity and *s* is a characteristic constants. For copper, *ρ*_0_ = 8.9* g*/*cm*^3^,* γ*_0_ = 2.02, *c*_0_ = 3.94 cm/μs, and *s* = 1.49 are used, while *ρ*_0_ = 1.0* g*/*cm*^3^,* γ*_0_ = 0.4934, *c*_0_ = 1.48 cm/μs, and *s* = 2.56 are used for water^35,36^. In order to be consistent with the theoretical model, a perfectly elastic and rigid plastic model is adopted to characterize solid with a constant shear modulus *G*_1_ and a constant yield strength *Y*.

The amplitude 2*ξ*_0_ is the distance from wave crest to trough in *y* direction. The length of the interface in *x* direction contains twenty wavelengths to diminish the effect of lateral extents. Square meshes with side length 2.5* μm* are distributed at initial moment. The solid plate is quiescent and the tangential velocity of the fluid plate is set to be *u*_0_ at the initial time.

## Amplitude motion equation by theory

For a two-dimensional configuration, the amplitude motion equation is derived starting from the governing equations of continuity and momentum without conservative and nonconservative forces acting on the interface

5 $$\nabla \cdot {{\varvec{u}}}_{i}=0\hspace{0.33em},$$

6 $${\rho }_{i}\left[\frac{\partial {{\varvec{u}}}_{i}}{\partial t}+\left({u}_{0,i}\cdot \nabla \right){{\varvec{u}}}_{i}\right]=-\nabla {p}_{i}\hspace{0.33em},$$

where *i* = 1 and *i* = 2 represents two materials respectively, **u**_*i*_ characterizes the irrotational perturbed velocity of material *i*, and *p*_*i*_ is pressure. Potential flow theory is adopted to establish the instability analysis. For irrotational flow at initial time and considering the tangential velocities, we have

7 $${{\varvec{u}}}_{i}=\nabla {\phi }_{i}\hspace{0.33em},$$

8 $${\Phi }_{i}={u}_{0,i}x+{\phi }_{i}\hspace{0.33em},$$

where *ϕ*_*i*_ and *Φ*_*i*_ satisfies Laplace equation. The mathematical formula of pressure can be obtained by integrating Eq. (6) from *y* = 0 to the instantaneous interface *y* = *η* (*x*, *t*) in *y* direction with *C*_1_ = *C*_2_^21^

9 $${p}_{i}=-\left({\rho }_{i}\frac{\partial {\phi }_{i}}{\partial t}+{\rho }_{i}{u}_{0,i}\frac{\partial {\phi }_{i}}{\partial x}+{C}_{i}\right).$$

With kinematic conditions in the normal direction

10 $$\frac{\partial \eta }{\partial t}=\nabla {\Phi }_{1}\cdot {\varvec{n}}=\nabla {\Phi }_{2}\cdot {\varvec{n}}$$

and potential function

11 $${\phi }_{i}={A}_{i}\left(t\right){e}^{\mp ky}{e}^{ikx}\hspace{0.33em},$$

we determine the coefficient *A*_*i*_(*t*) in terms of *ξ*(*t*) by considering the 2D coordinates system fixed on solid

12 $$\dot{\xi }\left(t\right)=-k{A}_{1}\left(t\right)=k{A}_{2}\left(t\right)-ik{u}_{0}\xi \left(t\right).$$

The force equilibrium is also acquired at the interface in normal direction

13 $${p}_{1}-{p}_{2}+{\sum }_{j}{{F}_{y}}^{\left(j\right)}=0\hspace{0.33em},$$

where *F*_*y*_^(*j*)^ represent force per unitary surface acting on interface. For system of EP solid and viscous fluid, the upper formula can be expressed as

14 $${p}_{1}-{S}_{1,yy}^{\left(ep\right)}-({p}_{2}-{S}_{2,yy}^{(v)})=0\hspace{0.33em},$$

where *S*_1,*yy*_^(*ep*)^ represents the vertical component of the deviatoric stress for EP solid and *S*_2,*yy*_^(v)^ is the vertical component of the deviatoric part of the Cauchy stress tensor *σ*_*ij*_ = -*pδ*_*ij*_ + *S*_*ij*_ for fluid.

The solid in elastic stage is assumed to be Hookean solid having linear constitutive relation^37^

15a $${\dot{S}}_{1,ij}=2{G}_{1}{D}_{1,ij}\hspace{0.33em},$$

15b $${D}_{1,ij}=\frac{1}{2}\left(\frac{\partial {u}_{1,i}}{\partial {x}_{j}}+\frac{\partial {u}_{1,j}}{\partial {x}_{i}}\right)\hspace{0.33em},$$

where *D*_1,*ij*_ is the strain rate tensor. The deviatoric stress tensors of fluid have the form of

16a $${S}_{2,ij}^{(v)}=2{\mu }_{2}{D}_{2,ij}\hspace{0.33em},$$

16b $${D}_{2,ij}=\frac{1}{2}\left(\frac{\partial {u}_{2,i}}{\partial {x}_{j}}+\frac{\partial {u}_{2,j}}{\partial {x}_{i}}\right)\hspace{0.33em}.$$

where *μ*_2_ is dynamic viscosity. The vertical component of the deviatoric stress for elastic solid and viscous fluid are obtained with Eqs. (7), (11), (12), (15a), (15b), (16a) and (16b)

17a $${S}_{1,yy}^{\left(ep\right)}=-2{G}_{1}k\left(\xi -{\xi }_{0}\right){e}^{ikx-ky}\hspace{0.33em},$$

17b $${S}_{2,yy}^{(v)}=2{\mu }_{2}k({\xi }^{^{\prime}}+ik{u}_{0}\xi ){e}^{ikx-ky}\hspace{0.33em}.$$

Then, the motion of amplitude for elastic equation can be described by Eq. (18) with substituting Eqs. (17a) and (17b) into Eq. (14)

18 $$\ddot{\xi }+\frac{2{\mu }_{2}{k}^{2}+2{\rho }_{2}{u}_{0}ki}{{\rho }_{1}+{\rho }_{2}}\dot{\xi }-\frac{{\rho }_{2}{u}_{0}^{2}{k}^{2}-2{\mu }_{2}{u}_{0}{k}^{3}i}{{\rho }_{1}+{\rho }_{2}}\xi =-\frac{2{G}_{1}{k}^{2}}{{\rho }_{1}+{\rho }_{2}}\left(\xi -{\xi }_{0}\right)\hspace{0.33em}.$$

The perturbation amplitude *ξ*_*p*_ at when solid yields is determined by the condition of EP transition, i.e. the effective stress $\tilde{\sigma }=\sqrt{3{S}_{1,ij}{S}_{1,ij}/2}$ arrives at *Y*. Then, the mathematical expression for plastic solid is derived with Eqs. (15a) and (15b) and with the fact that the plastic deformation only occurs at a small layer away form surface such as *y* ~ *k*^-1^. The integrated motion equation of amplitude for EP solid and viscous fluid is

19 $$\ddot{\xi }+\frac{2{\mu }_{2}{k}^{2}+2{\rho }_{2}{u}_{0}ki}{{\rho }_{1}+{\rho }_{2}}\dot{\xi }-\frac{{\rho }_{2}{u}_{0}^{2}{k}^{2}-2{\mu }_{2}{u}_{0}{k}^{3}i}{{\rho }_{1}+{\rho }_{2}}\xi -\left\{\begin{array}{c}\frac{2{G}_{1}{k}^{2}}{{\rho }_{1}+{\rho }_{2}}\left(\xi -{\xi }_{0}\right)\hspace{0.33em},\hspace{1em}\xi \le {\xi }_{p}\\ \frac{e}{\sqrt{3}}\frac{kY}{{\rho }_{1}+{\rho }_{2}}\hspace{0.33em}.\hspace{1em}\hspace{1em}\hspace{0.33em}\hspace{0.33em}\xi >{\xi }_{p}\end{array}\right.\hspace{0.33em}.$$

In order to focus on the instability on solid surface, viscosity effect of fluid is not discussed temporarily. The amplitude motion of KHI between EP solid and ideal fluid is described by taking *μ*_2_ = 0 in Eq. (19)

20 $$\ddot{\xi }+\frac{2{\rho }_{2}{u}_{0}ki}{{\rho }_{1}+{\rho }_{2}}\dot{\xi }-\frac{{\rho }_{2}{u}_{0}^{2}{k}^{2}}{{\rho }_{1}+{\rho }_{2}}\xi =-\left\{\begin{array}{c}\frac{2{G}_{1}{k}^{2}}{{\rho }_{1}+{\rho }_{2}}\left(\xi -{\xi }_{0}\right)\hspace{0.33em},\hspace{1em}\xi \le {\xi }_{p}\\ \frac{e}{\sqrt{3}}\frac{kY}{{\rho }_{1}+{\rho }_{2}}\hspace{0.33em}.\hspace{1em}\hspace{1em}\hspace{0.33em}\hspace{0.33em}\xi >{\xi }_{p}\end{array}\right.$$

whose dimensionless form is

21 $$\ddot{z}+2{M}_{1}i\dot{z}=\left\{\begin{array}{c}\Lambda -\left(\Lambda -{M}_{2}\right)z,\hspace{1em}\hspace{1em}z\le {z}_{p}\\ {M}_{2}z-{\rm X},\hspace{1em}\hspace{1em}\hspace{1em}\hspace{1em}\hspace{0.33em}z>{z}_{p}\end{array}\right.$$

with the definition of dimensionless variables *A*_*T*_ = (*ρ*_1_-*ρ*_2_) / (*ρ*_1_ + *ρ*_2_), *M*_0_^2^ = *ρ*_1_*u*_0_^2^/*G*_1_, *z* = *ξ*(*t*)/*ξ*_0_, *τ* = *tku*_0_, $\widehat{\lambda }$= 2*πξ*_0_/*λ* and $\widehat{Y}$= *ρ*_1_*u*_0_^2^/*Y*. *z*_*p*_ is growth factor at when EP transition takes place and

22 $${z}_{p}=\frac{\rm X}{\Lambda }+1,\hspace{1em}{M}_{1}=\frac{1-{A}_{T}}{2}\hspace{0.33em},\hspace{1em}{M}_{2}=\frac{1-{A}_{T}}{2}\hspace{0.33em},\hspace{1em}\Lambda =\frac{1+{A}_{T}}{{M}_{0}^{2}}\hspace{0.33em},\hspace{1em}{\rm X}=\frac{e}{2\sqrt{3}}\frac{1+{A}_{T}}{\widehat{\lambda }\widehat{Y}}\hspace{0.33em}.$$

$\widehat{\lambda }$ represents the characteristic of interface and $\widehat{Y}$ denotes the inducement and resistance on instability.

## Instability boundary by theory

The condition of stability is that the amplitude must have a maximum value at a certain time *τ* = *τ*_*m*_ which implies

23 $$\dot{z}({\tau }_{m})=0\hspace{0.33em},\hspace{1em}\hspace{1em}\ddot{z}({\tau }_{m})=0\hspace{0.33em}.$$

The deriving are started from Eq. (21) with initial conditions *z*(0) = 1 and *ż*(0) = 0. The transformations

24a $${x}_{1}=[\Lambda -(\Lambda -{M}_{2})z]{e}^{i{M}_{1}\tau },\hspace{1em}\hspace{1em}z\le {z}_{p}$$

24b $${x}_{2}=\left({M}_{2}z-{\rm X}\right){e}^{i{M}_{1}\tau }\hspace{0.33em},\hspace{1em}\hspace{1em}z>{z}_{p}$$

are introduced into Eq. (21) to achieve

25a $${\ddot{x}}_{1}=[-{M}_{1}^{2}-(\Lambda -{M}_{2})]{x}_{1}\hspace{0.33em},\hspace{1em}\hspace{1em}z\le {z}_{p}$$

25b $${\ddot{x}}_{2}=(-{M}_{1}^{2}+{M}_{2}){x}_{2},\hspace{1em}\hspace{1em}\hspace{0.33em}\hspace{0.33em}\hspace{1em}z>{z}_{p}$$

and the initial conditions and continuous conditions are

26a $${x}_{1}(0)={M}_{2}\hspace{0.33em},\hspace{1em}{\dot{x}}_{1}(0)=i{M}_{1}{M}_{2}\hspace{0.33em},$$

26b $${x}_{1}({\tau }_{p})={x}_{2}({\tau }_{p})={x}_{p}\hspace{0.33em},$$

26c $${\dot{x}}_{1}({\tau }_{p})={\dot{x}}_{1p},\hspace{1em}{\dot{x}}_{2}({\tau }_{p})={\dot{x}}_{2p},$$

where *τ*_*p*_ is the time when the solid transients from elasticity to plasticity. Integrating Eq. (25a) with Eq. (26a) and evaluating Eqs. (26b) and (26c) at time *τ*_*p*_ when the marginal stable state transits to plastic regime, it has

27 $${\dot{x}}_{1p}=-\sqrt{[-{M}_{1}^{2}-(\Lambda -{M}_{2})]({x}_{p}{)}^{2}+(\Lambda -{M}_{2}){M}_{2}^{2}}\hspace{1em}z\le {z}_{p}\hspace{0.33em},$$

Then, performing integration of Eq. (25a) twice with Eq. (26a) and (26b),we get

28 $${\tau }_{p}=-\frac{1}{\sqrt{-{M}_{1}^{2}-\left(\Lambda -{M}_{2}\right)}}\left[{\mathit{sin}h}^{-1}\left(\frac{{x}_{p}}{{M}_{2}}\sqrt{\frac{-{M}_{1}^{2}-\left(\Lambda -{M}_{2}\right)}{\Lambda -{M}_{2}}}\right)-{\mathit{sin}h}^{-1}\left(\sqrt{\frac{-{M}_{1}^{2}-\left(\Lambda -{M}_{2}\right)}{\Lambda -{M}_{2}}}\right)\right]\hspace{0.33em}.$$

By taking a first integration on Eq. (25b) with Eq. (26b) and (26c) and evaluating *x*_2_(*τ*_*m*_) = *ẋ*_2_(*τ*_*m*_) = 0 due to Eq. (23), we have

29 $${\dot{x}}_{2p}=-{x}_{p}\sqrt{-{M}_{1}^{2}+{M}_{2}}.$$

Then, by carrying the first derivatives of Eqs. (24a) and (24b) and evaluating them at *τ* = *τ*_*p*_, and combining Eqs. (27) and (29), we obtain *x*_*p*_

30 $${x}_{p}=-\frac{{M}_{2}^{2}}{\sqrt{(-2{M}_{1}^{2}+{M}_{2}+2i{M}_{1}\sqrt{-{M}_{1}^{2}+{M}_{2}})\Lambda }}.$$

Evaluating Eq. (26b) into Eq. (24a) and combining *z*_*p*_ in Eq. (22), it has

31 $$\frac{\rm X}{\Lambda }=\frac{{M}_{2}-{x}_{p}{e}^{-i{M}_{1}{\tau }_{p}}}{\Lambda -{M}_{2}}\hspace{0.33em},$$

With the definition of Χ in Eq. (22), the instability boundary is achieved

32 $${\widehat{Y}}_{IB}=\frac{e}{2\sqrt{3}}\frac{1+{A}_{T}}{\Lambda \widehat{\lambda }}\frac{\Lambda -{M}_{2}}{{M}_{2}-{x}_{p}{e}^{-i{M}_{1}{\tau }_{p}}}.$$

## EP division by theory

EP transition happens when the maximum amplitude *z*_*m*_^*e*^ = *z*(*τ*_*e*_) of pure elasticity becomes equal to the amplitude *z*_*p*_ for the occurrence of the plastic flow. From Eq. (24a), it has

33 $${x}_{1e}=[\Lambda -(\Lambda -{M}_{2}){z}_{m}^{e}]{e}^{i{M}_{1}{\tau }_{e}},$$

where *τ*_*e*_ is the time when EP transition takes place. Since *ż* (*τ*_*e*_) = 0, the derivative of Eq. (24a) at *τ* = *τ*_*e*_ is

34 $${\dot{x}}_{1e}={\dot{x}}_{1}({\tau }_{e})={M}_{1}{x}_{1e}i$$

and evaluating the first integration of Eq. (25a) at *τ* = *τ*_*e*_ , it has

35 $${x}_{1e}=-{M}_{2}\hspace{0.33em},$$

In order to obtain the transition time *τ*_*e*_, integrating Eq. (25a) twice and evaluating at *τ* = *τ*_*e*_ with Eq. (35), we get

36 $${\tau }_{e}=\frac{2}{\sqrt{-{M}_{1}^{2}-\left(\Lambda -{M}_{2}\right)}}{\mathit{sin}h}^{-1}\left(\sqrt{\frac{-{M}_{1}^{2}-\left(\Lambda -{M}_{2}\right)}{\Lambda -{M}_{2}}}\right)$$

Therefore, combining Eq. (33) with *z*_*m*_^*e*^ = *z*_*p*_, it has

37 $$\frac{\rm X}{\Lambda }=\frac{{M}_{2}\left(1+{e}^{-i{M}_{1}{\tau }_{e}}\right)}{\Lambda -{M}_{2}}$$

and using the definition of Χ again in Eq. (22), the expression of EP division is found

38 $${\widehat{Y}}_{EP}=\frac{e}{2\sqrt{3}}\frac{1+{A}_{T}}{\Lambda \widehat{\lambda }}\frac{\Lambda -{M}_{2}}{{M}_{2}\left(1+{e}^{-i{M}_{1}{\tau }_{e}}\right)} .$$

## Growth factor by theory

Growth factor are the solutions of Eq. (21) and the solving processes are similar to previous work^21^. Here, we list the dimensionless forms of the solutions. Two stable ones are

39a $$z(\tau )=\frac{1}{\Lambda -{M}_{2}}\{\Lambda -\frac{1}{2}{M}_{2}{e}^{-i{M}_{1}\tau }[(1+\frac{{M}_{1}}{\sqrt{{M}_{1}^{2}+(\Lambda -{M}_{2})}}){e}^{i\sqrt{{M}_{1}^{2}+(\Lambda -{M}_{2})}\tau }+(1-\frac{{M}_{1}}{\sqrt{{M}_{1}^{2}+(\Lambda -{M}_{2})}}){e}^{-i\sqrt{{M}_{1}^{2}+(\Lambda -{M}_{2})}\tau }]\}\hspace{0.33em}\hspace{0.33em},$$

for the purely elastic case below *z*_*p*_ and

39b $$z\left(\tau \right)=\left\{\begin{array}{c}\frac{1}{\Lambda -{M}_{2}}\{\Lambda -\frac{1}{2}{M}_{2}{e}^{-i{M}_{1}\tau }[(1+\frac{{M}_{1}}{\sqrt{{M}_{1}^{2}+\left(\Lambda -{M}_{2}\right)}}){e}^{i\sqrt{{M}_{1}^{2}+\left(\Lambda -{M}_{2}\right)}\tau }+(1-\frac{{M}_{1}}{\sqrt{{M}_{1}^{2}+\left(\Lambda -{M}_{2}\right)}}){e}^{-i\sqrt{{M}_{1}^{2}+\left(\Lambda -{M}_{2}\right)}\tau }]\}\hspace{0.33em},\hspace{1em}\tau \le {\tau }_{p}\\ \frac{1}{{M}_{2}}\{X+\frac{1}{2}{e}^{-i{M}_{1}\tau }[({x}_{p}+\frac{{\dot{x}}_{2p}}{\sqrt{-{M}_{1}^{2}+{M}_{2}}}){e}^{\left(\tau -{\tau }_{p}\right)\sqrt{-{M}_{1}^{2}+{M}_{2}}}+({x}_{p}-\frac{{\dot{x}}_{2p}}{\sqrt{-{M}_{1}^{2}+{M}_{2}}}){e}^{-\left(\tau -{\tau }_{p}\right)\sqrt{-{M}_{1}^{2}+{M}_{2}}}]\}\hspace{0.33em},\hspace{1em}\hspace{1em}{\tau }_{p}\le \tau \le {\tau }_{m}\\ {z}_{m}-\frac{X-{M}_{2}{z}_{m}}{\Lambda -{M}_{2}}\{1-\frac{1}{2}{e}^{-i{M}_{1}\left(\tau -{\tau }_{m}\right)}[(1+\frac{{M}_{1}}{\sqrt{{M}_{1}^{2}+\left(\Lambda -{M}_{2}\right)}}){e}^{i\left(\tau -{\tau }_{m}\right)\sqrt{{M}_{1}^{2}+\left(\Lambda -{M}_{2}\right)}}+(1-\frac{{M}_{1}}{\sqrt{{M}_{1}^{2}+\left(\Lambda -{M}_{2}\right)}}){e}^{-i\left(\tau -{\tau }_{m}\right)\sqrt{{M}_{1}^{2}+\left(\Lambda -{M}_{2}\right)}}]\}\hspace{0.33em},\hspace{1em}\hspace{1em}\hspace{1em}\hspace{1em}\tau \ge {\tau }_{m}\end{array}\right.$$

with EP transition. Two unstable solutions are, respectively,

39c $$z\left(\tau \right)=\left\{\begin{array}{c}\frac{1}{\Lambda -{M}_{2}}\{\Lambda -\frac{1}{2}{M}_{2}{e}^{-i{M}_{1}\tau }[(1+\frac{{M}_{1}}{\sqrt{{M}_{1}^{2}+\left(\Lambda -{M}_{2}\right)}}){e}^{i\sqrt{{M}_{1}^{2}+\left(\Lambda -{M}_{2}\right)}\tau }+(1-\frac{{M}_{1}}{\sqrt{{M}_{1}^{2}+\left(\Lambda -{M}_{2}\right)}}){e}^{-i\sqrt{{M}_{1}^{2}+\left(\Lambda -{M}_{2}\right)}\tau }]\}\hspace{0.33em},\hspace{1em}\tau \le {\tau }_{p}\\ \frac{1}{{M}_{2}}\{X+\frac{1}{2}{e}^{-i{M}_{1}\tau }[({x}_{p}+\frac{{\dot{x}}_{2p}}{\sqrt{-{M}_{1}^{2}+{M}_{2}}}){e}^{\left(\tau -{\tau }_{p}\right)\sqrt{-{M}_{1}^{2}+{M}_{2}}}+({x}_{p}-\frac{{\dot{x}}_{2p}}{\sqrt{-{M}_{1}^{2}+{M}_{2}}}){e}^{-\left(\tau -{\tau }_{p}\right)\sqrt{-{M}_{1}^{2}+{M}_{2}}}]\}\hspace{0.33em},\hspace{1em}\hspace{1em}\hspace{1em}\hspace{0.33em}\hspace{0.33em}\tau \ge {\tau }_{p}\end{array}\right.$$

and

39d $$z\left(\tau \right)=\left\{\begin{array}{c}\frac{1}{\Lambda -{M}_{2}}\{\Lambda -\frac{1}{2}{M}_{2}{e}^{-i{M}_{1}\tau }[(1+\frac{i{M}_{1}}{\sqrt{-{M}_{1}^{2}-(\Lambda -{M}_{2})}}){e}^{\tau \sqrt{-{M}_{1}^{2}-(\Lambda -{M}_{2})}}+(1-\frac{i{M}_{1}}{\sqrt{-{M}_{1}^{2}-(\Lambda -{M}_{2})}}){e}^{-\tau \sqrt{-{M}_{1}^{2}-(\Lambda -{M}_{2})}}]\}\hspace{0.33em},\hspace{1em}\tau \le {\tau }_{p}\\ \frac{1}{{M}_{2}}\{X+\frac{1}{2}{e}^{-i{M}_{1}\tau }[({x}_{p}+\frac{{\dot{x}}_{2p}}{\sqrt{-{M}_{1}^{2}+{M}_{2}}}){e}^{(\tau -{\tau }_{p})\sqrt{-{M}_{1}^{2}+{M}_{2}}}+({x}_{p}-\frac{{\dot{x}}_{2p}}{\sqrt{-{M}_{1}^{2}+{M}_{2}}}){e}^{-(\tau -{\tau }_{p})\sqrt{-{M}_{1}^{2}+{M}_{2}}}]\}\hspace{0.33em},\hspace{1em}\tau \ge {\tau }_{p}\end{array}\right.$$

## Supplementary Information

Supplementary Information.

## Data Availability

The datasets used and analyzed during the current study are available from the corresponding author on reasonable request.
