# Supplementary material for: Hydrodynamic Kelvin–Helmholtz instability on metallic surface
Source: Sci Rep. 2023 Feb 15;13:2686. doi: 10.1038/s41598-023-29810-7 (PMC9931697; doi:10.1038/s41598-023-29810-7)
Supplement: Supplementary file 1 — Supplementary Information. [file 41598_2023_29810_MOESM1_ESM.docx]

**Supplementary information**

Instability boundary and EP division by simulations in the paper are performed with the dimensionless form. Different dimensionless variables *A_T_*, *M*_0_, $\hat{\lambda}$ and $\hat{Y}$ in simulations are obtained by changing fluid density, tangential velocity, initial amplitude, wavelength, shear modulus and yield strength respectively. The specific values of each physical parameter of each point in Figs. 3(a), 3(b) and 3(c) in the paper are listed in Table 1.1, 1.2 and 1.3 respectively.

**Table 1.1 Simulation data of instability boundary and EP division for Fig. 3(a)**

| *ξ*_0_  (*μm*) | *λ*  (*μm*) | *u*_0_  (*mm*/*μs*) | *G*_1_  (*GPa*) | *Y_IB_*  (*MPa*) | *Y_EP_*  (*MPa*) | 2π*ξ*_0_/*λ* | *ρ*_1_*u*_0_^2^/*Y_IB_* | *ρ*_1_*u*_0_^2^/*Y_EP_* |
| --- | --- | --- | --- | --- | --- | --- | --- | --- |
| 10 | 250 | 1.0 | 39.38 | 158.42 | 304.92 | 0.251 | 56.179 | 29.188 |
| 10 | 160 | 1.0 | 39.38 | 244.90 | 459.78 | 0.393 | 36.342 | 19.357 |
| 10 | 500 | 1.0 | 39.38 | 82.42 | 157.46 | 0.126 | 107.978 | 56.522 |
| 10 | 800 | 1.0 | 39.38 | 54.50 | 99.96 | 0.079 | 163.317 | 89.036 |
| 13 | 160 | 1.0 | 39.38 | 317.19 | 553.87 | 0.511 | 28.059 | 16.069 |
| 15 | 200 | 1.2 | 56.71 | 423.53 | 796.87 | 0.471 | 30.260 | 16.083 |
| 11 | 200 | 1.2 | 56.71 | 309.68 | 573.91 | 0.346 | 41.385 | 22.331 |
| 10 | 300 | 1.2 | 56.71 | 194.87 | 369.73 | 0.209 | 65.766 | 34.663 |
| 10 | 600 | 1.2 | 56.71 | 102.44 | 187.47 | 0.105 | 125.109 | 68.364 |
| 14 | 210 | 0.8 | 25.20 | 169.41 | 314.92 | 0.419 | 33.622 | 18.087 |
| 10 | 210 | 0.8 | 25.20 | 124.80 | 219.55 | 0.299 | 45.641 | 25.945 |
| 10 | 380 | 0.8 | 25.20 | 67.41 | 134.81 | 0.165 | 84.501 | 42.251 |

**Table 1.2 Simulation data of instability boundary and EP division for Fig. 3(b)**

| *ξ*_0_  (*μm*) | *λ*  (*μm*) | *u*_0_  (*mm*/*μs*) | *G*_1_  (*GPa*) | *Y_IB_*  (*MPa*) | *Y_EP_*  (*MPa*) | 2π*ξ*_0_/*λ* | *ρ*_1_*u*_0_^2^/*Y_IB_* | *ρ*_1_*u*_0_^2^/*Y_EP_* |
| --- | --- | --- | --- | --- | --- | --- | --- | --- |
| 10 | 250 | 0.8 | 39.38 | 327.48 | 624.00 | 0.251 | 17.393 | 9.128 |
| 10 | 160 | 0.8 | 39.38 | 509.80 | 874.28 | 0.393 | 11.173 | 6.515 |
| 10 | 500 | 0.8 | 39.38 | 164.85 | 319.69 | 0.126 | 34.553 | 17.817 |
| 10 | 800 | 0.8 | 39.38 | 108.48 | 199.50 | 0.079 | 52.508 | 28.551 |
| 13 | 160 | 0.8 | 39.38 | 646.15 | 1147.83 | 0.511 | 8.815 | 4.962 |
| 15 | 200 | 0.96 | 56.71 | 847.06 | 1548.39 | 0.471 | 9.683 | 5.297 |
| 11 | 200 | 0.96 | 56.71 | 646.15 | 1147.83 | 0.346 | 12.694 | 7.146 |
| 10 | 300 | 0.96 | 56.71 | 384.42 | 734.69 | 0.209 | 21.337 | 11.164 |
| 10 | 600 | 0.96 | 56.71 | 204.88 | 389.74 | 0.105 | 40.035 | 21.045 |
| 14 | 210 | 0.64 | 25.20 | 349.71 | 595.83 | 0.419 | 10.424 | 6.118 |
| 10 | 210 | 0.64 | 25.20 | 249.60 | 454.51 | 0.299 | 14.605 | 8.021 |
| 10 | 380 | 0.64 | 25.20 | 144.83 | 264.91 | 0.165 | 25.171 | 13.761 |

**Table 1.3 Simulation data of instability boundary and EP division for Fig. 3(c)**

| *ξ*_0_  (*μm*) | *λ*  (*μm*) | *u*_0_  (*mm*/*μs*) | *G*_1_  (*GPa*) | *Y_IB_*  (*MPa*) | *Y_EP_*  (*MPa*) | 2π*ξ*_0_/*λ* | *ρ*_1_*u*_0_^2^/*Y_IB_* | *ρ*_1_*u*_0_^2^/*Y_EP_* |
| --- | --- | --- | --- | --- | --- | --- | --- | --- |
| 10 | 250 | 0.5 | 39.38 | 382.48 | 768.83 | 0.251 | 5.817 | 2.894 |
| 10 | 160 | 0.5 | 39.38 | 634.64 | 1047.62 | 0.393 | 3.506 | 2.124 |
| 10 | 500 | 0.5 | 39.38 | 194.87 | 403.46 | 0.126 | 11.418 | 5.515 |
| 10 | 800 | 0.5 | 39.38 | 124.80 | 249.60 | 0.079 | 17.829 | 8.914 |
| 13 | 160 | 0.5 | 39.38 | 847.06 | 1247.99 | 0.511 | 2.627 | 1.783 |
| 15 | 200 | 0.6 | 56.71 | 1047.62 | 1794.44 | 0.471 | 3.058 | 1.786 |
| 11 | 200 | 0.6 | 56.71 | 746.67 | 1348.15 | 0.346 | 4.291 | 2.377 |
| 10 | 300 | 0.6 | 56.71 | 473.68 | 904.31 | 0.209 | 6.764 | 3.543 |
| 10 | 600 | 0.6 | 56.71 | 239.58 | 483.71 | 0.105 | 13.373 | 6.624 |
| 14 | 210 | 0.4 | 25.20 | 409.76 | 724.14 | 0.419 | 3.475 | 1.966 |
| 10 | 210 | 0.4 | 25.20 | 289.66 | 553.87 | 0.299 | 4.916 | 2.571 |
| 10 | 380 | 0.4 | 25.20 | 164.85 | 334.93 | 0.165 | 8.638 | 4.252 |
